# Lebrikizumab for atopic dermatitis in the elderly: A case series

**DOI:** 10.1016/j.jdcr.2026.05.012

**Published:** 2026-05-11

**Authors:** Barbara Gruber

**Affiliations:** Department of Dermatology and Venereology, Klinikum Wels-Grieskirchen, Wels, Austria

**Keywords:** atopic dermatitis, elderly population, IL-13 inhibitor, IL-13 pathway, lebrikizumab

## Introduction

Atopic dermatitis (AD) is a chronic, relapsing inflammatory skin disorder characterized by eczematous lesions,^1^ associated with an increased risk of secondary skin infections (commonly presents with intense pruritus), which significantly contributes to sleep disturbances, psychosocial burden and reduced quality of life.^2^ While AD is predominantly diagnosed in early childhood and often clears after puberty, it can persist into mid-to-late adulthood or emerge as late-onset AD, affecting up to 12% of the elderly population.^1^

Among this elderly population, the classic AD could be presented by chronic pruritic, xerotic, lichenified eczematous plaques, often involving flexures, neck, eyelids, hands, and trunk, with marked skin dryness related to age-associated barrier dysfunction. Moreover, atypical AD could be presented by facial erythema, pigmented neck eczema, prurigo nodularis or lichenified lesions in flexural sites of the extremities.^1^^,^^3^ In some cases, skin biopsy is essential to confirm AD and exclude mimickers, as mycosis fungoides that can closely resemble chronic AD in this age group.^4^

In these patients, skin directed therapy remains the foundation of AD management, particularly for mild-to-moderate disease. This includes regular use of emollients to address age related xerosis, along with topical anti-inflammatory treatments such as corticosteroids and calcineurin inhibitors.^3^ However, mobility limitations, caregiver dependence, and cognitive impairment may have a significant impact on therapeutic adherence in this age group.^5^ Additionally, older adults face an increased risk of adverse events due to higher rates of polypharmacy. The management approach followed by the European guidelines does not include specific recommendations tailored to elderly patients.^6^ Biological therapies, such as dupilumab and tralokinumab, have shown AD improvement in skin lesions and itch in adult as well as in elderly patients with moderate-to-severe AD, with non-serious adverse effects.^7^, ^8^, ^9^ Lebrikizumab (LEB) a biological therapy, has demonstrated long-term efficacy and safety in individuals with moderate-to-severe AD,^10^^,^^11^ and to be significantly more efficacious than placebo on patients aged 60 years and older at week 16.^12^ However, further evidence is required to confirm the efficacy and long-term safety of LEB in older adults, a population that remains underrepresented in both epidemiologic studies of AD and clinical trials evaluating novel treatments.^13^

In the following series, we present 3 AD cases in elderly patients that experienced profound improvement after treatment with LEB, with real-world data obtained at a dermatological outpatient clinic practice in Austria between December 2022 and March 2025.

## Case descriptions

### Case 1

A 62-year-old male patient with a history of chronic AD in the forearms and lower legs, along with occasional joint pain and pollinosis visited the dermatology clinic in December 2022. Initial treatment included local corticosteroids and narrow band ultraviolet B radiation (UVB) phototherapy. Due to persistent symptoms, he was screened for systemic treatment with a biological agent.

**Fig 1:**
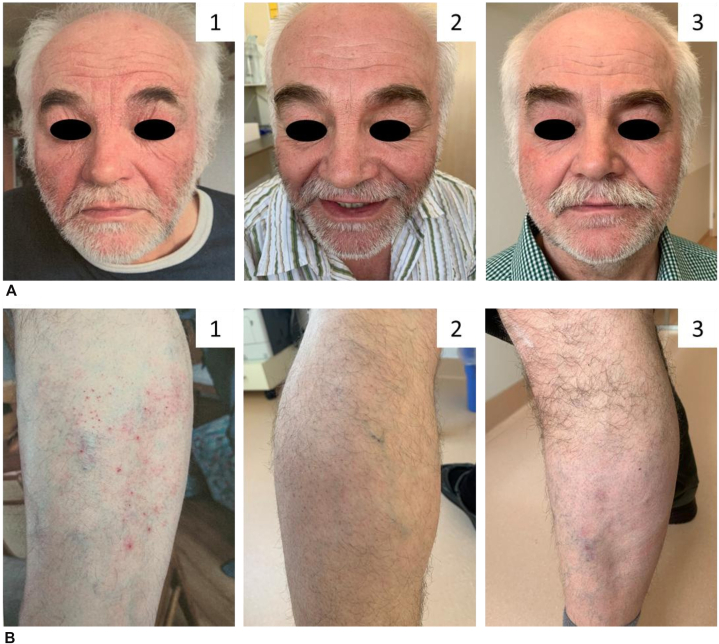
Skin evolution of a 62-year patient with severe AD (case study 1) treated with lebrikizumab, at baseline (1), week 4 (2) and at week 47 (3). This figure includes the following anatomic areas: **(A)** Face: 1 (baseline)—erythematous face with dry, flaky patches around the eyebrows and the eyes, 2 (week 4)—very residual scaling localized in the cheeks, and sides of the nose, and 3 (week 47)—completely clear face. **B,** Lower leg: 1 (baseline)—lower leg with erythematous papules and macules, excoriation marks and dry texture, 2 (week 4)—lower leg with post-inflammatory hyperpigmentation, mild scaling, mild erythema, and 3 (week 47)—completely clear lower leg.

Treatment: The patient was initiated with oral upadacitinib treatment at a daily dosage of 30 mg. Following 4 weeks of treatment, the patient reported significantly less itching. Continued therapy over 6 months led to complete resolution of itching, and unremarkable laboratory results. At the 7-month mark, the patient was diagnosed with diverticulitis, prompting the discontinuation of upadacitinib. After treatment termination the patient initially remained symptom-free but experienced a gradual deterioration over the next 7 months, eventually presenting to the clinic in April 2024 with an AD exacerbation significantly affecting the face and lower legs (Fig 1, *A1* and *B1*). The skin was assessed as Investigator's Global Assessment (IGA) score 4 and the patient reported a score of 10 on the itch-numerical rating scale (NRS). Treatment with LEB was initiated, following a 16-week induction period with 1 subcutaneous injection (250 mg) twice per week in weeks 0 and 2, and then 1 injection every 2 weeks. After 4 weeks of treatment with LEB treatment, most facial lesions had cleared (IGA 0) and the patient reported a vast improvement of pruritus (itch-NRS 2) and high level of treatment satisfaction (Fig 1, *A2* and *B2*). At week 14, coinciding with a seasonal pollen exposure, the patient presented with an acute pollinosis along with an exacerbation of skin symptoms (IGA 1) and a generalized increase in pruritus (itch-NRS 4), though without associated sleep disturbances; nevertheless, LEB treatment was continued according to protocol. At week 24, the patient exhibited an Eczema Area and Severity Index (EASI) 75 response and a complete resolution of pruritus and was switched to the recommended maintenance dose (250 mg every fourth week). By week 35, the patient exhibited stable skin condition, sustained itch relief and reported high treatment satisfaction. The patient remained symptom free (IGA 0, itch-NRS 0) through week 47 (Fig 1, *A3* and *B3*) up until the last visit at week 58. During which the patient reported that he did not experience a pollinosis induced AD exacerbation of this season.

### Case 2

**Fig 2:**
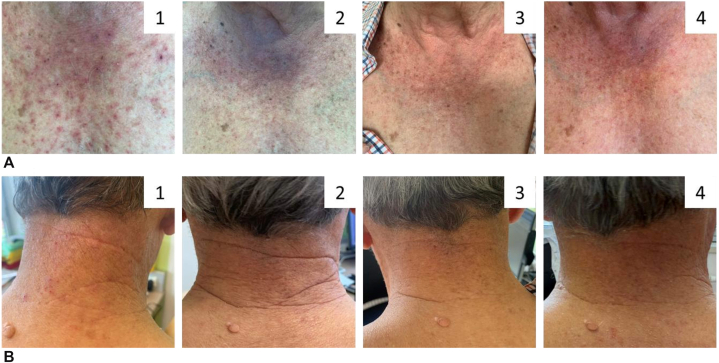
Skin evolution of an 88-year patient with severe AD (case study 2) treated with lebrikizumab, at baseline (1), week 16 (2), week 24 (3), and week 45 (4), including the following anatomic areas: **(A)** Décolleté: 1 (baseline)—numerous erythematous papules and small crusted lesions, 2 (week 16)—mild and diffuse erythema, 3 (week 24)—mottled post-inflammatory hyperpigmentation over faint background erythema, and 4 (week 45) —faint background erythema. **B,** Back of the neck: 1 (baseline)—patchy erythema, fine scaling, lichenified lines and hypopigmented areas around the lines in the neck, 2 (week 16)—reduced lichenification and completely healed lesions, 3 (week 24)—skin on the neck showing sustained remission with no lichenification, eczema nor patchy dry areas, and 4 (week 45)—sustained response.

An 88-year-old male patient with severe AD (IGA 4) presented at the dermatology clinic in July 2024 with very intense pruritus (itch-NRS 10) affecting the arms, legs, neck and décolleté, which significantly impaired sleep (Fig 2, *A1* and *B1*). In the past, pruritus consistently affected the periorbital area as well. The patient had previously been treated with various advanced and conventional systemic therapies, including tralokinumab (discontinued due to secondary loss of efficacy), dupilumab (discontinued due to secondary loss of efficacy), and upadacitinib (discontinued due to thoracic pain), and immunosuppressants such as cyclosporine, azathioprine, and methotrexate.

Treatment: The patient initiated LEB therapy following the 16-week induction period. By week 12, a significant improvement in skin symptoms was noted (IGA 2, EASI 50), and the patient reported the complete resolution of pruritus (itch-NRS 0) accompanied by an improved sleep quality. Lebrikizumab treatment was continued in the induction period as per protocol. At week 16, further clinical improvement was observed, including complete resolution of the AD on the neck and reduced diffuse erythema in the décolleté (Fig 2, *A2* and *B2*). At this timepoint, the skin symptoms were assessed as IGA 1 with an EASI 75 improvement, and the dosing interval was adjusted to 1 injection every 4 weeks (maintenance dose). Continued therapy over 24 weeks showed a sustained absence of pruritus, and a complete resolution of erythema and lichenification in the back of the neck (IGA 0; EASI 90) (Fig 2, *A3* and *B3*). At week 37, the patient was experiencing a slight disease flare (IGA 1, itch-NRS 2) after an unrelated infection. At the most recent visit (week 45), the patient presented an IGA 1 and reported a resolution of pruritus (itch-NRS 0) (Fig 2, *A4* and *B4*).

### Case 3

**Fig 3:**
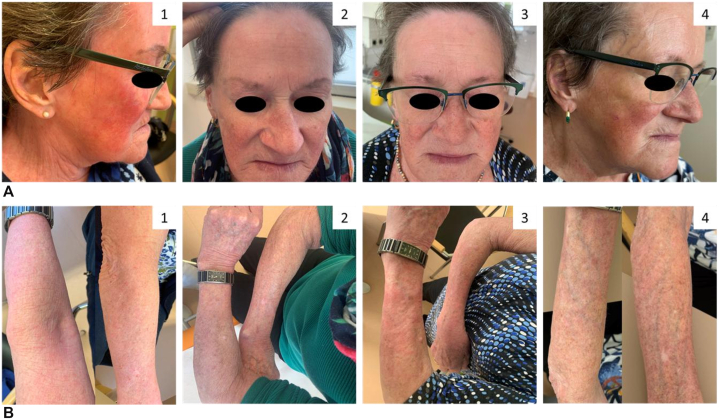
Skin evolution of an 73-year patient with severe AD (case study 3) treated with lebrikizumab, at baseline (1), week 5 (2), week 13 (3), week 29 (4), including the following anatomic areas: **(A)** Arms: 1 (baseline)—faint scaling and small excoriations and papules overlying a lichenified right arm and a lichenified left arm, both with thickened skin, 2 (week 5)—diffuse erythema and mild xerosis over both forearms without obvious signs of lichenification, 3 (week 13)—slight thickening of the skin and diffuse erythema extending along the forearms, and 4 (week 29) a complete clearance of lignification and erythema. **B,** Face: 1 (baseline)—prominent erythema over the cheek and jawline of the face with highly lichenified skin on the neck, 2 (week 5)—mild erythema and fine scaling on the lateral face, forehead, cheeks and periorbital region and very residual scaling around the nose area, 3 (week 13)—mild erythema over the cheeks and around the eyes with localized dryness around the perioral and periorbital areas, and 4 (week 29) a complete clearance of lignification on the neck and face, with rosacea localized on the cheeks.

In November 2024, a 73-year-old female patient presented severe AD (IGA 4) involving the head and face with dry, inflamed, erythematous areas, intense pruritus (itch-NRS 10) localized to the face and neck, and a lichenified left lower arm (Fig 3, *A1* and *B1*). Previous treatments included upadacitinib 15 mg, which was discontinued due to loss of efficacy and inability to increase the dosage due to safety concerns, baricitinib (discontinued due to transaminase increase) and lastly dupilumab, which was discontinued due to lack of efficacy in reducing pruritus.

Treatment: The patient was initiated on LEB according to the 16-week induction period. At week 5, the patient demonstrated a significant clinical improvement under LEB therapy, achieving an IGA score of 2 and an EASI 50 response. There was a marked reduction in facial erythema, decreased pruritus (itch-NRS 5) and a significant improvement of sleep quality (Fig 3, *A2* and *B2*). At week 13, the patient experienced an acute flare of a chronic, severe pollen allergy, presenting ocular symptoms, axillary skin involvement, pruritus, and sleep disturbances. However, the facial skin remained unaffected (Fig 3, *A3* and *B3*). At that time, the patient was assessed with an IGA score of 1-2 (almost clear – mild disease) and exhibited an EASI 75 response. Additional symptomatic treatment was initiated, including antihistamines and local therapy. Lebrikizumab therapy was continued at the induction period dosing schedule of 1 subcutaneous injection every 2 weeks until week 16, at which point the patient was switched to a 4-week dosing. At week 29, the patient presented a complete clearance of erythema on the arms and of lichenification on the arms, neck and face with AD unrelated rosacea on the cheeks (IGA 1, EASI 90 response, and resolution of pruritus [itch-NRS 0]) (Fig 3, *A4* and *B4*).

## Discussion

AD in the elderly poses unique diagnostic and therapeutic challenges due to its atypical presentation, comorbidities, polypharmacy and altered immune responses associated with aging, reducing treatment tolerability, effectiveness and adherence. We describe 3 cases of moderate-to-severe AD in older patients with a history of inadequate response or intolerance to multiple systemic therapies, who were successfully treated with subcutaneous LEB. Lebrikizumab, a recently authorized treatment for moderate-to-severe AD, has shown robust efficacy in clinical trials^10^^,^^11^ that was later confirmed in a post-hoc analysis restricted to the older population.^12^ Currently real-world evidence in this clinically relevant group of patients is limited. This case report addresses this gap by demonstrating consistent improvement in this underrepresented age group following the initiation of LEB.

All patients reported reductions in pruritus (as early as week 4), with sustained therapeutic responses. Two patients with preexisting allergies reported, transient AD exacerbations coinciding with seasonal pollen exposure, an established environmental trigger in patients with IgE-mediated sensitization to environmental allergens.^3^ These flare-ups manifested as intensified pruritus and were effectively managed with symptomatic therapy without interrupting the LEB treatment. Interestingly, 1 of the 2 patients reported that he did not experience a pollinosis induced exacerbation of the AD in his second allergy season while on LEB. It is worth noting that LEB targets the same type 2 inflammation pathway involved in AD, which is also responsible for pollinosis-associated symptoms, potentially offering a dual beneficial effect.

By week 16, all patients had achieved EASI 75 and IGA 1 responses. Two patients reached EASI 90 response by week 24 and week 29, respectively. One patient achieved complete AD resolution (IGA 0, NRS 0) by week 35. Notably, prior therapies such as dupilumab, Janus kinase (JAK) inhibitors and conventional immunosuppressants had been discontinued due to either lack of response or adverse events.

This case series represents real-world clinical evidence on the use of LEB for moderate-to-severe AD in the elderly population. All cases tolerated LEB well, with no treatment-related adverse events reported. Therefore, these findings suggest that LEB may be a promising therapeutic option for elderly patients with refractory AD, including those with prior biologic or JAK inhibitor failure. Despite some cases showed early significant improvement, LEB requires 16 weeks for full efficacy. Treatment with LEB may take longer than others to reach its peak effect, but it offers better long-term control. Further studies involving larger cohorts of elderly patients with AD and longer follow-up are needed to confirm long-term safety and efficacy of LEB in this age group.

In conclusion, this real-world evidence demonstrates sustained clinical improvement observed in older patients (>60 years) with moderate-to-severe AD treated with LEB. These cases suggest that LEB may be a well-tolerated treatment with a positive response for elderly patients, including those with a history of inadequate response to previous systemic AD treatments.

## Conflicts of interest

Gruber has received consulting fees from Abbvie, Almirall, Amgen, Eli-Lilly, Janssen-Cilag, Leo Pharma, Novartis and Pfizer.
